# Stable, efficient, and cost-effective system for the biosynthesis of recombinant bacterial cellulose in Escherichia coli DH5α platform

**DOI:** 10.1186/s43141-022-00384-7

**Published:** 2022-06-23

**Authors:** Saif S. Al-Janabi, Heba Shawky, Amr A. El-Waseif, Ayman A. Farrag, Tarek M. Abdelghany, Dina E. El-Ghwas

**Affiliations:** 1 Botany and Microbiology Department, Faculty of Science (Boys), Al-Azhar University, Cairo, Egypt; 2 Department of Medical Laboratory Techniques, Al-Maarif University College, Al-anbbar, Iraq; 3 Therapeutic Chemistry Department, Pharmaceutical and Drug Industries Research Institute, National Research Centre, Dokki 12622, Cairo, Egypt; 4 Chemistry of Natural and Microbial Products Department, Pharmaceutical and Drug Industries Research Institute, National Research Centre, Dokki, Cairo, 12622 Egypt

**Keywords:** Bacterial cellulose, Molecular cloning, *E.coli* DH5α, Biosynthesis, Plasmid stability

## Abstract

**Background:**

Owing to its remarkable mechanical properties that surpass the plant-based cellulose, bacterial cellulose production has been targeted for commercialization during the last few years. However, the large-scale production of cellulose is generally limited by the slow growth of producing strains and low productivity which ultimately makes the commercial production of cellulose using the conventional strains non cost-effective. In this study, we developed a novel plasmid-based expression system for the biosynthesis of cellulose in *E.* *coli* DH5α and assessed the cellulose productivity relative to the typically used *E.* *coli* BL21 (DE) expression strain.

**Results:**

No production was detected in BL21 (DE3) cultures upon expression induction; however, cellulose was detected in *E.* *coli* DH5α as early as 1 h post-induction. The total yield in induced DH5α cultures was estimated as 200 ± 5.42 mg/L (dry weight) after 18 h induction, which surpassed the yield reported in previous studies and even the wild-type *Gluconacetobacter* *xylinum* BRC5 under the same conditions. As confirmed with electron microscope micrograph, *E.* *coli* DH5α produced dense cellulose fibers with ~ 10 μm diameter and 1000–3000 μm length, which were remarkably larger and more crystalline than that typically produced by *G.* *hansenii*.

**Conclusions:**

This is the first report on the successful cellulose production in *E.* *coli* DH5α which is typically used for plasmid multiplication rather than protein expression, without the need to co-express *cmcax* and *ccpAx* regulator genes present in the wild-type genome upstream the *bcs*-operon, and reportedly essential for the biosynthesis.

## Background

Bacterial cellulose (BC) has been recently a hallmark of several important biomedical products including vessel implants (tubes), wound care dressings, surgical threads, biocompatible implants, tissue reinforcement matrices besides other conventional industries like paper, food products, electronics, and cosmetic products [1]. As it was found to be privileged over plant-based cellulose in terms of renewability and biodegradability besides its unique mechanical properties including higher crystallinity; purity, water holding capacity, biocompatibility, and elasticity [2]; BC has been an attractive target for commercial production in recent years.

**Fig. 1:**
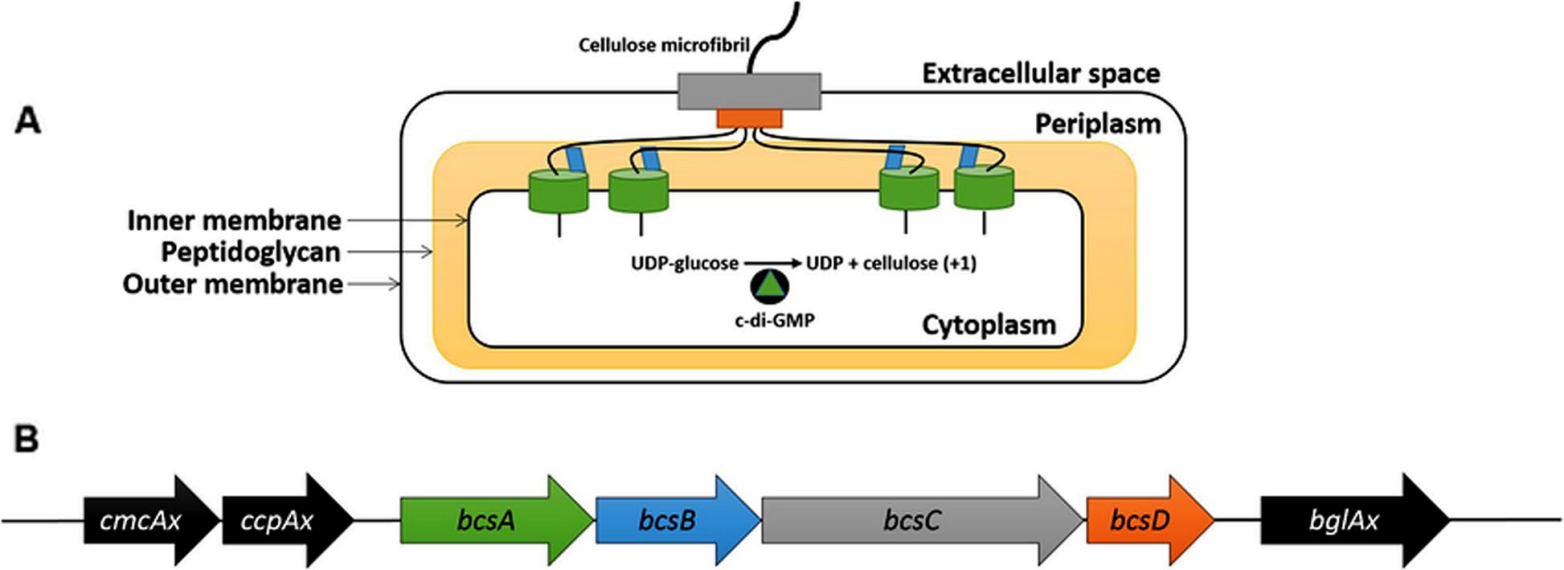
Genetic organization of *bcs* operon [3]. **A** When *bcs*A (**green**) is activated by c-di-GMP, it incorporates glucose units into a cellulose chain in the cytoplasm using UDP glucose as a substrate. *BcsB* (**blue**) guides the glucan chain through the periplasm; *bcs*D (**orange**) crystallizes four glucan chains in the periplasm, and finally, *bcs*C (**gray**) exports the BC micro-fibrils into the extracellular space. **B** The genetic organization of the *bcs* operon encoding the bacterial cellulose synthase complex, with regulatory *cmcax*, *ccpAx* genes upstream *bgIAx* gene downstream the operon. The genes in the *bcs* operon (**B**) are color-matched with their protein products (**A**). This work is licensed under the Creative Commons Attribution 4.0 International License. To view a copy of this license, visit http://creativecommons.org/licenses/by/4.0/ or send a letter to Creative Commons, PO Box 1866, Mountain View, CA 94,042, USA

In cellulose-producing bacterial strains, most prominent of which is *Gluconoacetobacter* *hansenii*, BC biosynthesis is orchestrated by four genes; namely A, B, C, and D comprising a 9.2-kb-long bacterial cellulose synthase (bcs) operon, preceded by two “regulatory” fragments reportedly essential for BC biosynthesis; *cmcax* gene that encodes an endo-β-1,4-glucanase enzyme that hydrolyzes BC and improves its synthesis; and *ccpAx* gene believed to play an important role in extracellular transportation of BC (Fig. 1) [3, 4]. The main BC synthase (GDP-forming) enzyme (PF03552) is encoded by the *bcs*A fragment, while *bcs*B encodes a regulatory protein that belongs to the BcsB superfamily (pfam03170) encoding cellulose biosynthesis regulatory protein thought to bind the cyclic di-GMP-positive effector [5]; both comprise the complex glycosyl transferase unit responsible for transferring glucosyl residues from UDP glucose to the 1,4-glucan chain [6]. Meanwhile, the fragments *bcs*C (InterPro: IPR001440) and D (InterPro: IPR022798) are believed to be implicated in extracellular exporting and packaging of cellulose fibrils by forming a channel to the outer membrane allowing BC crystallization [7]. Several studies have reported that only *bcs*AB fragments are enough for effective in vitro BC production [8]; however, other studies confirmed the necessity of the four genes for maximum in vivo production [9].

The mass production of BC in different plasmid-based systems has been addressed in several studies [8, 10, 11]. In this domain, *E.* *coli* platforms have been extensively used as superior protein producers for their rapid growth kinetics, ease of genetic manipulation, and rapid protein expression among other privileges [12]. Among the most widely used bacterial hosts for recombinant proteins mass production are *E.* *coli* B strains [Rosetta and BL21 (DE3)] and K-12 strains including the main strain of plasmid replication DH5α [13]. However, as a membrane-associated protein, cellulose synthase overexpression was reported to be toxic in *E.* *coli* BL21 (DE3), which paved the way for other alternative hosts like C41 (DE3); a mutant strain derived from BL21 (DE3) to study the possibility of mass expression of toxic membrane proteins of *bcs*-operon [11]. In a previous report, we managed to amplify, clone, and characterize the nucleotide/amino acid sequences of the four *bcs*-ABCD fragments from *Gluconoacetobacter* *hansenii* using a new approach of PCR amplification [14]. Herein, we propose and assess the efficiency and applicability of this novel approach in terms of a successful production of BC in the *E.* *coli* (DH5α) platform for the first time and compare the BC productivity with the typically used *E.* *coli* BL21 (DE3) expression strain.

## Methods

### Screening of positive transformants

The cloning efficiency of *bcs* amplicons was confirmed by colony PCR on individual transformants grown on 2XYT/carbencillin agar plates. As previously reported [14], every two genes were cloned in one backbone to simplify the subsequent transformation into *E.* *coli* competent cells. Each insert was individually screened for correct orientation downstream the T5 promoter using both vector primers flanking the insert and the specific amplification primers of each fragment as listed in (Table 1) below. PCR reactions were carried out in SimpliAmp gradient thermal cycler (**Thermofisher Scientific, USA**) using Platinum™ SuperFi II DNA Polymerase (**Invetrogen™—USA**). The reaction mixture included 100 ng of purified plasmid template, 10 μl buffer, 200 mM final concentration of each primer (Table 1), 1 μl of dNTP mix, 1 μl of SuperFi II DNA Taq polymerase enzyme, 0.6 μl DMSO, and the reaction volume was completed to 50 μl with free-nuclease water. The cycling conditions included one cycle of pre-denaturation for 30 s at 98 °C, followed by 35 cycles of denaturing at 98 °C for 10 s, annealing at 60 °C (universal temperature of Platinum™ SuperFi II DNA Polymerase) for 10 s (universal annealing temp of SuperFi II polymerase), extension for 30 s/kb at 72 °C, and a final extension cycle for 10 min at 72 °C. PCR products were resolved on agarose gel (0.7–1% according to the amplicon size) premixed with 0.5 μg/ml ethidium bromide, and the size of the resolved products was determined with 1 kb DNA ladder (**Thermofisher Scientific, USA**). The gel was run at 80 V for 20 min followed by visualization on a gel documentation system (**Biometra, Goettingen, Germany**). The transformed colonies bearing correctly oriented inserts were then cultured in liquid broth and subjected to plasmid purification using GeneJET Plasmid Miniprep Kit (**Thermofisher Scientific, USA**) according to the user manual. Purified clones of *bcs*AB and *bcs*CD were co-transformed into competent *E.* *coli* DH5α and BL21 (DE3) by the heat-sock method according to Sambrook et al. [15].

**Table 1:** List of primers used for screening of *bcsAB* and *bcsCD-*positive transformants for correct orientation downstream T5 promoter in pQE-Trisystem expression vectors

Clone	Gene ID	Primer	Nucleotide sequence
***bcs*AB**	***bcs*A**	**pQE-F**	5′- GTTATTGTGCTGTCTCATC-3′
		***bcs*A-R**	3′-CGAGGCCGCACGGCTGATCTT-5′
	***bcs*B**	***bcs*B-F**	5′-AAAATGGTGTCCCTGATCGCGCTG-3′
		**pQE-R**	5′-ATCGATCTCAGTGGTATTTGTGA-3′
***bcs*CD**	***bcs*C**	**pQE-F**	5′- GTTATTGTGCTGTCTCATC-3′
		***bcs*C** **R**	3′-CTGGTCCATAATAAGATAGTGTGCGGAAATCA-5′
	***bcs*D**	***bcs*D-F**	5′-ACAACTTTGAACGCAAAACCGGACTTTTCG-3′
		**pQE-R**	5′-ATCGATCTCAGTGGTATTTGTGA-3′

### Monitoring growth rate and plasmid stability

The bacterial growth rate of both *E.* *coli* strains was spectrophotometrically monitored by measuring the optical density of overnight cultures grown at a temperature range of 22–37 °C at 600 nm. Plasmid stability over the same temperature range was assessed according to Buldum et al. [11] with minor modifications, where overnight cultures grown at each temperature were used to seed fresh LB/2XYT media supplemented with 50 mg/mL carbencillin, then allowed to grow at the same temperature till the mid-log phase (OD_600_ ~ 0.5–0.6). 1/1000 dilution of each culture was plated on selective (with antibiotic) and free (without antibiotic) LB/2XYT agar plates. The colony-forming units (CFU) were counted after cultivation at the same growth temperature overnight, and plasmid stability was calculated as the percentage of plasmid-harboring colonies grown on selective plates/total viable colonies on free plates.

### Optimization of protein expression conditions

Several conditions including the selection of culture media, pre/post-induction incubation temperature, the final concentration of isopropyl *b*-D-thiogalactoside (IPTG), and incubation time have been assessed. Individual cloned *E.* *coli* DH5α/BL21 (DE3) colonies were grown to saturation at different incubation temperatures ranging between 22 and 37 °C in 10 ml LB, 2XYT, and TB culture media containing 100 μg/ml carbencillin. One milliliter of these starter cultures was inoculated into 100 ml of 100 μg/ml carbencillin-containing media and cultured with shaking at 180 rpm until OD_600_ reaches 0.8–1.2. Expression of recombinant was induced by IPTG to a final concentration of 0.01–0.2 mM. After a 5-h induction at 22 °C and 180 rpm, 1 mL of induced cells was harvested by centrifugation at 4 °C and 5000 × *g* for 30 min and stored at − 20 °C for SDS profiling, and the rest was used for recovery of bacterial cellulose.

### Profiling of recombinant bcs proteins

One milliliter of induced cultures was centrifuged, and the pellet was first washed with nuclease-free water twice and then was mixed with 2 × sample buffer and boiled for 5 min to denature the proteins. The 11–245 kDa marker and the samples were loaded into the wells of 14% SDS polyacrylamide gels. Separation of proteins based on size was achieved by allowing them to run at 120 V for approximately 90 min [16].

### Purification of recombinant bacterial cellulose

BC-producing cultures were first filtered to recover the BC particles. The filtered BC was treated with 1% NaOH solution (w/v) at 70 °C for 20 min to remove bacteria and other impurities, then rinsed with deionized water several times until reaching a neutral pH, and then dried in micro-centrifuge tubes at 50 °C to constant weight to obtain dry weight [11].

### Characterization of recombinant BC by scanning electron microscope (SEM)

Before analysis, recombinant BC was dried until constant weight is reached and placed onto double-sided carbon tape mounted onto an aluminum stub. They were gold-coated for 2 min at 20 mA using an **Emitech K575X Peltier (Ashford, UK)** cooled sputter coater. The morphological and microstructural features of recombinant BC were assessed using field-emission scanning electron microscope (FESEM) LEO 1525 (Zeiss, Germany) operating at 5 kV [11].

### Statistical analysis

All numerical results were analyzed for unpaired parametric *t* test, Pearson correlation, and non-linear regression (curve fit) analysis using GraphPad Prism version 9.0.2 (GraphPad, San Diego, CA). Samples in each assay were assayed in triplicates. Results of all assays are corrected and expressed as mean ± SD. *P* values < 0.05 were considered statistically significant.

## Results

### Screening for correctly oriented inserts in positive transformants

**Fig. 2:**
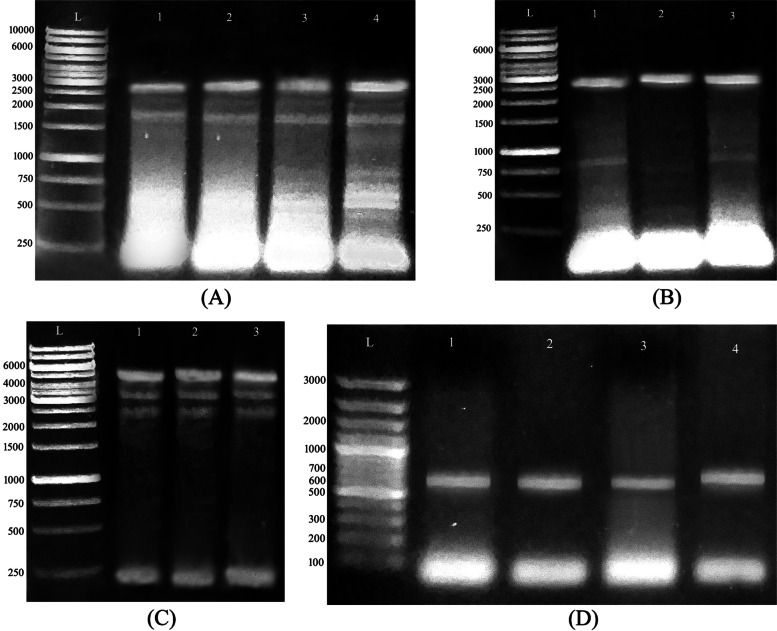
Screening of *bcs*-encoding fragments in individual *E.* *coli* DH5α/BL21 (DE3) transformed colonies using vector/amplification primers. Three-four colonies were selected in each plate for PCR-screening, and the individual fragments *bcs*A (**A**), *bcs*B (**B**), *bcs*C (**C**), and *bcs*D (**D**) were detected in all of the tested transformants at M. wt of ~ 2.43 kb, 2.6 kb, 4.1 kb, and 672 bp, respectively. Lane (L) refers to Generuler1kb plus DNA ladder

The cloned individual fragments were visualized at the expected molecular weight for each gene in all tested colonies, where *bcs*A and B inserts were detected at M. wt of ~ 2.431 (2.2 kb insert size plus 231 bp distance from the forward vector primer) (Fig. 2A) and 2.6 kb (2.4 kb insert size plus 201 bp distance from the vector primer), respectively (Fig. 2B), while *bcs*C and D were visualized at ~ 4.130 kb (3.9 kb insert size plus 231 bp distance from the forward vector primer) (Fig. 2C) and 671 bp (471 bp insert size plus 201 bp distance from the vector primer), respectively (Fig. 2D), which confirmed successful cloning and co-transformation of both clones.

### Effect of culture conditions on growth kinetics and plasmid stability

**Fig. 3:**
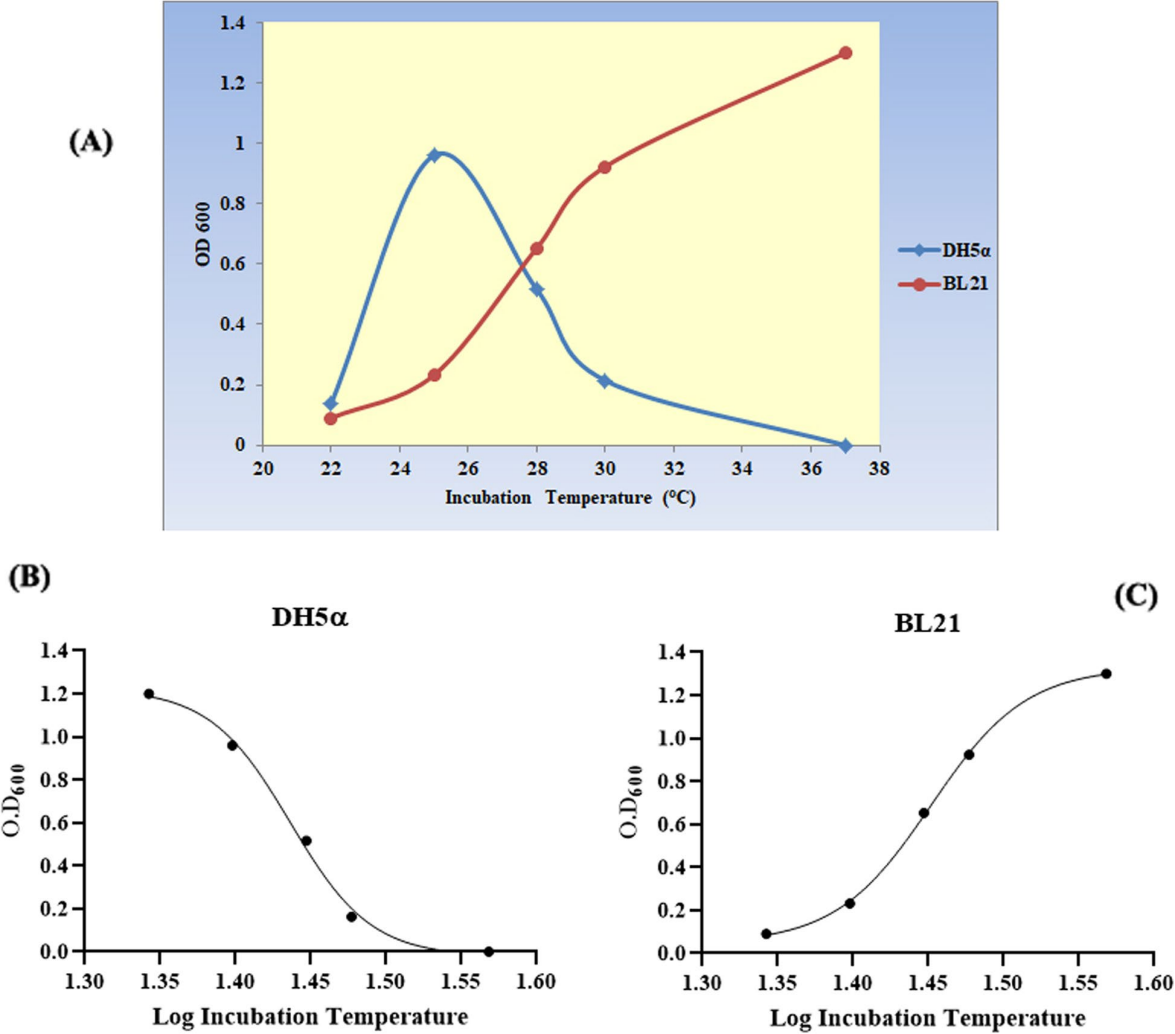
The growth kinetics of recombinant *E.* *coli* DH5α and BL21 (DE3) (**A**). Cultures of *E.* *coli* DH5α showed higher growth density and rate as incubation temperature declined, achieving the highest growth rate at 25 °C. Non-linear regression analysis is demonstrated to show the inverse correlation (*r* =  − 0.9383, *P* = 0.0091) between growth rate and temperature. Unlike DH5α, cultures of BL21 (DE3) showed a positive correlation with growth temperature (*r* = 0.9779, *P* = 0.002) (**B**), where they grew faster in elevated temperatures reaching the maximum at 37 °C (**C**)

Both *E.* *coli* strains showed differential growth kinetics over the tested culture media and the range of incubation temperatures, where the density of DH5α cultures as measured in terms of optical density at 600 nm tended to be lower at elevated temperatures, and the optimum growth was observed at 25 °C (OD_600_ = 0.962), unlike those of BL21 (DE3) that showed higher densities as the incubation temperature increased, with the maximum culture density observed at 37 °C (OD_600_ = 1.42) (Fig. 3A). The growth of recombinant *E.* *coli* strains was also found to be better in LB relative to 2XYT or TB media, in which no growth was observed at any incubation temperature (*P* = 0.001). The correlation between growth kinetics of both strains with incubation temperatures was statistically assessed, and results revealed strong significant correlations either inverse like in the *E.* *coli* DH5α model (*r* =  − 0.8392, *P* = 0.0451) (Fig. 3B) or positive like that observed in *E.* *coli* BL21 (DE3) model (*r* = 0.9779, *P* = 0.002) (Fig. 3C). The growth rates of recombinant *E.* *coli* DH5α were observed to be generally slower than BL21 under the same conditions. The pattern of plasmid stability over different growth temperatures followed the same of DH5α growth kinetics, where the highest was observed at a growth temperature of 22 °C (100%) while the lowest was observed at 30 °C (56%). On the other side, BL21 cultures showed a dramatic decline of plasmid stability; despite the dense bacterial growth, as the growth temperature elevated.

### Expression of recombinant bcs proteins and production of BC

**Fig. 4:**
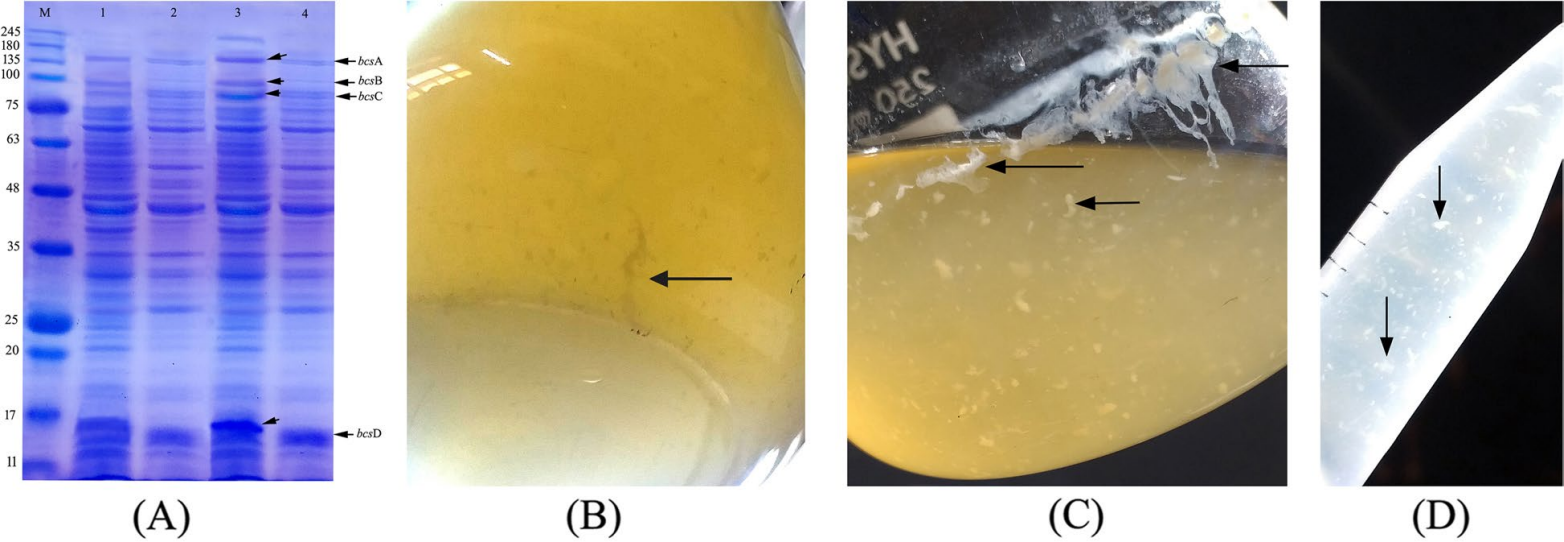
SDS-PAGE (14%) showing the profile of resolved recombinant *bcs*-proteins in induced cultures of *E.* *coli*. The four *bcs*-recombinant proteins were detected as the expected molecular weights in both B21 (DE3) and DH5α induced cultures (lanes 3 and 4, respectively), but their overexpression was more pronounced in later compared to control uninduced cultures of B21 (DE3) and DH5α (lane 1 and 2, respectively) (**A**). BC was detected as white gel-like dispersed structures in *E.* *coli* DH5α 1 h after IPTG induction (**B**), and it continued to accumulate in culture media until it was visualized as white filaments 18 h post-induction (**C**). A less dense BC production was detected in uninduced DH5α cultures suggesting a leaky expression (**D**)

For a preliminary assessment of our expression system, we followed the design that was previously proposed by Buldum et al. [11] with minor modifications. First, a recombinant colony from both *E.* *coli* DH5α and BL21 (DE3) was grown at the optimum temperature (25 °C and 37 °C, respectively) till the early stationary phase was reached as determined by OD_600_ ~ 0.96–1. Protein expression was initiated with 0.02-mM final concentration of IPTG for both strains, and post-induction incubation temperature was set for both at continued for 5 h at 22 °C. The four *bcs*-recombinant proteins were detected as the expected molecular weights in both induced cultures, but their overexpression was more pronounced in B21 (DE3) relative to DH5α cell lysates, in which no difference could be detected between induced and control culture profile. Given that BC is extracellularly expressed, it can be directly visualized in culture medium when the expression is successful. Despite the overexpression of cloned *bcs*-fragments as detected in SDS-PAGE (Fig. 4A), no production was visualized in BL21 (DE3) culture. Meanwhile, the cellulose production was detected in DH5α culture as white gel-like dispersed as early as 1 h post-induction (Fig. 4B), and expression continued over time till the expressed BC was visualized as white filaments after 5 h post-induction (Fig. 4C). A less dense production was also detected in the un-induced control culture (Fig. 4D), reflecting a basal expression typical to the constitutive vector even in the presence of high-glucose concentration that was reduced as a result of consumption as a primary carbon source during cell growth. The yield of BC in this system was calculated to be 12 mg/L, i.e., 33.4 mg/g. glucose/h after 5 h of IPTG induction, and elevated to reach ~ 200 ± 5.42 mg/g. glucose/h after overnight expression (18 h).

### Verification and preliminary characterization recombinant BC by scanning electron microscope

**Fig. 5:**
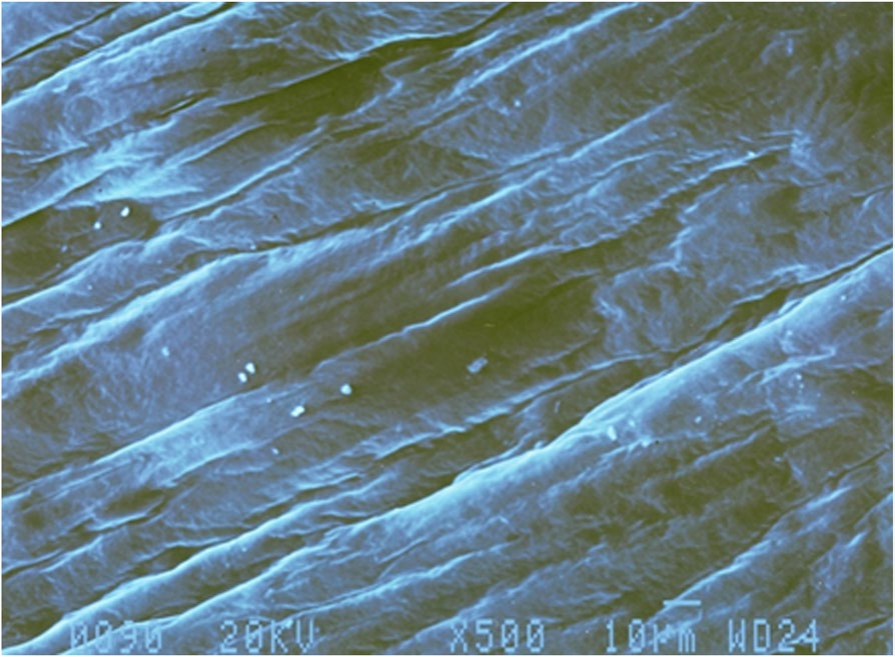
Scanning electron micrograph showing the morphology and microstructure of recombinant BC produced by *E.* *coli* DH5α at 500 times magnification

As confirmed by scanning electron micrograph, the *E.* *coli* DH5α strain produced dense BC fibers of ~ 10 μm in diameter and 1000–3000 μm in length, which are markedly larger than the cellulose produced by the wild-type strain *G.* *hansenii*. The recombinant BC membrane showed a white color due to the lower water content as shown in Fig. 5. The dried BC membrane surfaces showed network structure formed by aggregates of extended crystalline cellulose chains in an ultrafine network structure consisting of long nanofibers. Scanning electron microscopy showed that the crystallinity was slightly higher than that obtained from commercial sugars when used as a carbon source for wild-type growth.

## Discussion

The challenges facing the mass production of bacterial cellulose (BC) are mainly related to the characteristics of the producing strains which include, slow growth kinetics relative to many other bacteria (such as *E.* *coli*) as it requires at least 5 days to grow, low productivity of cellulose, and extreme susceptibility to changes culture/growth conditions that might generate spontaneous mutations in the BC-producing strains rendering them non-producing mutants besides the relatively large quantity of glucose/L required in the growth media for efficient cellulose production [17]. Even in the context of developing methods for the biosynthesis of BC using the typical protein expression strain *E.* *coli* BL21 (DE3), the expression of the periplasmic *bcs* enzyme was found to have a toxic effect on the host strain, as it disrupts its vital functions of the cellular membrane [18]. When another strain of *E.* *coli* C41 (DE3) which can produce proteins of potential toxicity was used, the overall production of BC was significantly lower than that of the wild-type *G.* *hansenii* [11]. In this report, we aimed to avoid all of the abovementioned limitations and employed a consecutive T5 promoter-based expression vector to allow the expression in *E.* *coli* strains other than the T7-dependant BL21 (DE3). To simplify the bacterial transformation, every two genes were cloned in one DNA backbone to investigate the possibility of efficient co-expression of two open-reading frames under the power of a single promoter. The two recombinant *bcs*AB and *bcs*CD vectors were successfully co-transformed in individual colonies of both *E.* *coli* strains with transformation efficiency approaching 100% as evident by the PCR screening results of positive transformants, where each fragment was visualized at the expected M. wt. However, the number of *bcs*AB clones of both DH5α and BL21 (DE3) strains was found to be generally lower than *bcs*CD, suggesting a basal/leaky expression of the periplasmic catalytic fragment of BC synthase which might impact the bacterial growth; a typical feature of the consecutive expression vector.

In general, the introduction of a recombinant plasmid into *E.* *coli* cells is bound to impose some metabolic load on the host cells for plasmid maintenance and replication, regardless of the nature of the foreign gene [19]. One of the main parameters that affect the stability of recombinant plasmids and thereby the bacterial growth kinetics is culture conditions [17, 18, 20]. In this domain, the effect of culture media composition on successful cloning and expression of recombinant proteins has been controversial, where some studies reported that culture media enriched with tryptone and yeast extract enhances plasmid stability and cellular growth rates [21], while others reported the opposite [17, 18]. Growth characteristics and stability of cloned plasmid are greatly influenced by incubation temperature as well [22]. Although *E.* *coli* has long been classified as a “mesophilic” bacterium with an optimum growth temperature of 37 °C [23], it has been later reported that elevated temperatures generally promote plasmid instability and thereby reduce growth rates, which compromises the whole process of protein production in *E.* *coli*-based expression systems [24]. Herein, the growth of recombinant *E.* *coli* colonies tended to be better in the low complexity media. In addition, the growth rates of recombinant *E.* *coli* DH5α was observed to be generally slower than BL21 under the same conditions, which can be explained by the reported sensitivity of DH5α to high-glucose concentration in the culture media, that was primarily used as a carbon source for cellulose production besides controlling pre-induction leaky expression, unlike BL21 that is less sensitive to glucose concentrations due to its active glyoxylate shunt and anaplerotic pathways and thereby can grow to higher densities [25].

Results also revealed that recombinant plasmid was generally more stable in DH5α cultures compared to BL21, which is typical given that DH5α is genetically engineered to be *end*A and *rec*A-deficient, i.e., lacking the fragment encoding endonuclease I (*end*A) that degrades double-stranded DNA and recombinase (*rec*A) that catalyzes homologous recombination by DNA strand exchange reactions [25, 26], besides the low copy number of pQE-Trisystem vector (20–30 copies per cell) that contributes the stability by reducing the metabolic burden of maintenance and segregation [27]. The pattern of plasmid stability over different growth temperatures followed the same of DH5α growth kinetics, where the highest was observed at a growth temperature of 22 °C while the lowest was observed at 30 °C, while it showed a dramatic decline as the growth temperature elevated in BL21 cultures despite the dense bacterial growth. This could be attributable to an escalating metabolic burden induced by elevated temperatures that might promote the basal expression of membrane-bound/periplasmic reportedly be toxic to the host cells [18].

Although the four *bcs*-recombinant proteins were visualized as the expected molecular weights in induced cultures of both *E.* *coli* cultures as revealed in SDS-PAGE, the overexpression was more pronounced in B21 (DE3) compared to DH5α cell lysates, which might be expected as *E.* *coli* DH5α is not typically engineered for protein expression [13]. However, no BC production was detected in BL21 (DE3) cultures despite the overexpression of cloned *bcs*-fragments, suggesting that either the recombinant proteins were accumulated into inactive inclusion bodies despite the low incubation temperature and IPTG final concentration or that IPTG induction has aggravated the metabolic stress by the overexpression of toxic membrane proteins that eventually lead to plasmid loss as previously proposed in previous reports [28]. In DH5α-induced cultures, the BC productivity was estimated as 200 ± 5.42 mg/L after overnight (18 h) induction, which surpassed the yield reported in previous studies of plasmid-based BC expression [11], and even the wild-type *Gluconacetobacter* *xylinum* BRC5 that produced 0.69 mg/g glucose/h in agitated conditions [29]. Also, SEM analysis results revealed that recombinant BC fibers had markedly higher crystallinity and larger diameter and length relative to the cellulose produced by the wild-type strain *G.* *hansenii*.

Altogether, and contradicting with the findings of Buldum et al. [11], these results prove that successful in vitro production of BC does not necessitate the introduction of *cmcax* and *ccpAx* genes upstream the *bcs*-operon and that the catalytic *bcs*AB region besides the *bcs*CD responsible for exporting cellulose extracellularly is enough for successful BC production in plasmid-based expression systems, which complies with previous studies conducted by Omadjela et al. [8] and the more recent findings proposed by Acheson et al. [30]. Indeed, the expression of BC in DH5α has been previously approached [31]; however, the level of the BC production was not examined as the study focused on the production in *Enterobacter* sp., which makes this study the first systematic report on BC production in *E.* *coli* DH5α without the upstream *cmcax* and *ccpAx* genes. Compared to the conventional production using wild-type cellulose-producing strains, the proposed system is more efficient in terms of the time of production, yield, and stability and more cost-effective as lower amounts of glucose (2-mM final concentration of glucose compared to 24 gm/L in conventional systems) required for cellulose biosynthesis. The system developed herein shows promising potentials for future bioprocess design for BC production on a commercial scale.

## Conclusions

In this report, we developed a new stable, efficient plasmid-based expression system of recombinant bacterial cellulose in *E.* *coli* DH5α platform. The recombinant biosynthesis of BC was successfully achieved without the need to co-express the upstream *cmcax* and *ccpAx* fragments reportedly essential for BC biosynthesis. Moreover, the constructed system proved efficient in producing BC with its remarkable fibrous structure in a yield that surpassed that of previous reports and the wild type in agitated conditions within 5 h after induction with low IPTG concentration. The full optimization of expression conditions for maximizing the BC production is beyond the scope of this report that primarily focused on assessing the efficiency of our novel amplification and cloning approach; yet, the system developed herein showed promising potentials for cost-effective production of bacterial cellulose, considering that this system requires lower concentrations of glucose and IPTG expression inducer.

### Limitations of this study

Results of this report would have been better in terms of BC productivity if DH10β strain was used, particularly that it is more suitable for large plasmids which would have enabled simultaneous cloning of the four genes in a single plasmid; avoiding potential problems of preferential transformation/expression of the two *bcs*-AB and *bcs*-CD clones co-administrated into single DH5α cells, not to mention the metabolic load of their large size (~ 20 kb/combined). However, as this work did not receive any funds and it was mostly supported by the personal fund, we were limited with the bacterial strains available in our lab and could not manage to purchase another strain.

## Data Availability

Data that support the findings of this study are available from the corresponding author upon reasonable request.

## Abbreviations

BC: Bacterial cellulose
*bcs*: Bacterial cellulose synthase
kb: Kilobase
bp: Base pair
IPTG: Isopropyl *b*-D-thiogalactoside
kDa: Kilodalton
M.wt: Molecular weight
